# Call for papers for a special issue on screening and prevention of communicable diseases in newly arrived migrants in Europe

**DOI:** 10.2807/1560-7917.ES.2017.22.13.30500

**Published:** 2017-03-30

**Authors:** Eurosurveillance editorial team

**Affiliations:** 1 European Centre for Disease Prevention and Control (ECDC), Stockholm, Sweden

*Eurosurveillance* invites authors to submit papers for a special issue on effectiveness and cost-effectiveness of screening and prevention of infectious diseases among newly arrived migrants in Europe.

According to the office of the United Nations High Commissioner for Refugees, the levels of displaced people are currently the highest ever recorded worldwide (http://www.unhcr.org/afr/figures-at-a-glance.html).

Since 2015, Europe has experienced an unprecedented increase in the numbers of newly arrived migrants. For example between 2015 and early 2016, over 1.2 million people arrived in Europe. In 2016 alone a total of 355,361 people arrived by sea in countries belonging to the European Union (EU), landing on the coasts of the Mediterranean sea. Over 900 were officially reported to have died or gone missing during their journey (http://data2.unhcr.org/en/situations/mediterranean#_ga=1.123760719.43188132.1490861483).

Reasons for migration are diverse and include seeking refuge and/or asylum from conflict, violence and persecution.

While most migrants are healthy, some sub-groups of migrants are disproportionally affected by certain diseases. The overwhelming share of health problems for newly arrived migrants in Europe are non-communicable diseases and chronic conditions along with mental disorders, and malnutrition. There is a debate among public health experts as to what screening and prevention measures should be offered to migrants to maintain a high level of public health in general and to improve the health of the newly arrived migrants to the EU/ European Economic Area in particular.

In order to contribute to the current body of evidence, the special issue on effectiveness and cost-effectiveness of screening and prevention of infectious diseases among newly arrived migrants in Europe, *Eurosurveillance* aims to provide new insights and aspects that could spark scientific debate and support decision making.

Topics of interest may include, but are not limited to, the following diseases/conditions/subjects:

Active and latent tuberculosis
HIV/AIDS
Hepatitis B
Hepatitis C
Vaccine-preventable diseases
Intestinal parasites
Feasibility, acceptability and cost effectiveness

The submission deadline is **1 July 2017**. If you would like to submit a paper or ask for more information, please see our instructions for authors regarding article formats and contact the editorial team at eurosurveillance@ecdc.europa.eu.

